# Social disparities, navigational health literacy, and unmet needs in Germany: results of a population survey

**DOI:** 10.3389/ijph.2026.1609928

**Published:** 2026-09-14

**Authors:** Till Werner Ulrich, Jens Klein, Olaf von dem Knesebeck

**Affiliations:** Institute of Medical Sociology, University Hospital Hamburg-Eppendorf, Hamburg, Germany

**Keywords:** Germany, healthcare access, navigational health literacy, social disparities, unmet needs

## Abstract

**Objectives:**

Navigational health literacy is increasingly important in the German health system. While social disparities have been reported, less is known about its association with unmet needs and whether it attenuates the associations between social characteristics and unmet needs.

**Methods:**

This cross-sectional online survey in Germany included a random sample of 2,201 adults. Participants completed the validated HLS19-NAV instrument and reported unmet needs during the past 12 months. Associations were examined using descriptive and multiple Poisson regression analyses.

**Results:**

Overall, 70% of participants showed inadequate navigational health literacy. Lower navigational health literacy was associated with lower income, lower education, statutory health insurance, and age 41–59 years. Unmet needs were more common among younger adults, women, and lower-income or statutorily insured participants. Better navigational health literacy was associated with lower prevalence of unmet needs. After adding navigational health literacy to the model, associations between social characteristics and unmet needs remained largely unchanged.

**Conclusion:**

Navigational health literacy was unequally distributed across groups and strongly associated with unmet needs but did not reduce the observed social disparities in access to care.

## Introduction

Due to its continuous expansion, the German healthcare system has become increasingly complex and challenging to navigate [1–6]. Consequently, the demands on users regarding orientation and navigation within the system have also increased. To address these challenges, navigational health literacy is required, which is defined as *“people’s knowledge, motivation, and skills to access, understand, appraise, and apply the information and communication in various forms necessary for navigating healthcare systems and services adequately to get the most suitable healthcare for oneself or related persons”* [7]. Although Rima Rudd and colleagues introduced a similar concept of healthcare navigation already in 2004 [8], research on navigational health literacy remains limited. As part of the European Health Literacy Survey 2019–2021 (HLS19) [9], the HLS19-NAV instrument was developed to measure navigational health literacy across countries. It was applied and validated in eight different countries, including Germany as part of the second Health Literacy Survey (HLS-GER2) [6, 7, 10, 11]. The HLS-GER2 study found that a substantial proportion of the German population exhibits low navigational health literacy (68.4% classified as inadequate, 14.4% as problematic, 8.9% as sufficient, and only 8.4% as excellent), leading to difficulties navigating within the healthcare system [6, 10, 11]. Furthermore, some studies have shown low navigational health literacy among individuals with lower subjective social status, advanced age, and lower level of education [6, 10, 12]. International HLS19 data show that challenges in navigational health literacy are not limited to Germany. Across the eight countries applying the HLS19-NAV, mean scores varied considerably, ranging from 41.6 in Germany and 48.6 in Belgium to 64.2 in Portugal, 66.8 in Austria, and 67.4 in Slovenia, indicating substantial cross-country differences in the ability to navigate healthcare systems [9]. However, these differences should be interpreted cautiously due to variation in survey modes and data collection periods across countries [9, 11]. Socioeconomic differences in navigational health literacy were also observed internationally, with financial deprivation and self-perceived social status identified as significant predictors in seven of the eight participating countries [11].

Unmet needs is a multidimensional concept and an indicator of access to care, broadly referring to situations in which individuals perceive a need for healthcare but do not receive, delay, or forgo care [13–15]. Unmet needs can be assessed using either clinical or subjective approaches: clinical approaches rely on professional assessments of whether appropriate care was received, whereas subjective approaches capture individuals’ own perceptions that they did not receive the care they needed [14, 15]. Such needs may arise from different barriers, including affordability, availability, accessibility, and acceptability of services [13]. In this study, we focus on self-reported unmet needs due to three non-chosen system-related barriers: financial costs, geographical availability, and waiting times [15].

According to the Organisation for Economic Co-operation and Development (OECD), an average of approximately 28% of adults who required healthcare within a 12-month period reported having forgone care due to financial costs (16%), delayed or forgone care due to long waiting times (18%), or travel distance (4%) in EU and OECD countries [16].

Research indicates that levels of unmet needs vary across European healthcare systems [16]. The proportion ranged from 10% in Norway to 47% in Ireland, with values exceeding 40% in Estonia, Iceland, Latvia, Portugal, and Ireland; in Germany, the proportion was above average at 32% [16]. Across EU and OECD countries, unmet needs were more common among lower-income groups [16]. However, reported levels of unmet need vary between surveys and countries as a result of differing methods and approaches [16, 17]. Germany features a distinct health insurance model, in which health insurance is mandatory and structured as a dual system, consisting of statutory health insurance and substitutive private health insurance. Approximately 11% of the population are covered by private health insurance, which is often associated with benefits such as shorter waiting times compared to statutory health insurance coverage [18].

Studies on forgone care in Germany have reported varying levels of unmet needs but consistently revealed social inequalities in access to care [19–24]. Although unmet needs is a well-established measure in health services research [16, 25], there remains a lack of research on how forgone care relates to navigational health literacy. Individuals with limited navigational health literacy may encounter difficulties in navigating the system and accessing appropriate care. These difficulties may include incorrect assumptions of financial costs, arranging appropriate appointments, or uncertainty about which healthcare provider to consult. Since navigational health literacy is itself socially patterned [6, 10, 12], it may also represent one pathway through which social disadvantage is linked to barriers in healthcare access. Therefore, it is important to examine whether navigational health literacy is correlated with unmet needs. While prior research has addressed various aspects of navigational health literacy, to our knowledge, this is the first study to examine its association with unmet needs. Furthermore, it examines whether navigational health literacy reduces social disparities in unmet needs. Therefore, the following research questions were addressed [1]: Are there social disparities in navigational health literacy? [2] Are navigational health literacy and unmet needs associated? [3] Does navigational health literacy attenuate the associations between social characteristics and unmet needs?

## Methods

### Study design and sample

Analyses were based on a cross-sectional online survey conducted by the social research institute forsa in November and December 2022. A random sample of 5,619 German-speaking adults (age ≥18 years) was selected from the forsa panel of approximately 150,000 individuals and invited to complete the survey via email. This panel was recruited offline via telephone, including both landline and mobile numbers. The panel is a population-based, representative sample of the adult population living in Germany who reported using the internet. After three reminders, N = 2,201 individuals participated. We anticipated that approximately 11% of the respondents to be covered by private health insurance [18]. As we also aimed to examine differences in healthcare access based on health insurance status, we targeted a sample size of approximately 2,200.

The sample was weighted for age, sex, federal state, and education (using the iterative proportional fitting approach [26]), according to the official statistics provided by the Federal Statistical Office of Germany [27]. Thus, the weighted sample adequately represents the adult population in Germany regarding these socio-demographic characteristics. The survey was approved by the Local Psychological Ethics Committee at the Center for Psychosocial Medicine, University Medical Center Hamburg (No. LPEK-0563).

### Measures

The following characteristics of the respondents were considered to reflect social disparities: age, sex, migration history, income, education, and health insurance. Age was categorized into three groups: 18–40 years, 41–59 years, and ≥60 years. Regarding migration history, respondents were categorized into three groups: people who have immigrated themselves (1st generation migrants); people who were born in Germany, but whose parents (one or both) have immigrated (2nd generation migrants), and those without a migration history. Monthly net household income was equivalized to consider household size and composition. It was then divided into quartiles. Educational level was assessed using the established CASMIN (Comparative Analysis of Social Mobility in Industrial Nations) educational classification, a hierarchically structured measurement of certificates including the general and vocational qualifications [28]. The nine original CASMIN-levels were merged into four educational groups: low (levels 1a, 1b and 1c), intermediate (2a and 2b), high (2c_gen and 2c_voc), and highest (3a and 3b). Health insurance was stated as either statutory or private.

Navigational health literacy was measured with the HLS19-NAV [6, 7]. It includes 12 items asking participants to indicate on a four-point rating scale (“very easy,” “easy,” “difficult,” “very difficult”) how easy/difficult it is to access (three items), understand (three items), appraise (three items), and apply (three items) information about navigational issues in the healthcare system [7, 11]. The instrument demonstrated high internal consistency in this sample, with a Cronbach’s α of 0.92. The answers were dichotomized to “very easy/easy” and “very difficult/difficult” for analysis and converted into a score (0–100) which reflects the percentage of answers given as “easy” or “very easy” [6]. In line with previous research, only participants who provided valid responses to at least 10 of the 12 items, the minimum whole number required to meet the predefined 80% threshold, were included in the score, resulting in 242 missing values for navigational health literacy (see Table 1) [6, 7]. The score was divided into four categories according to previous studies (excellent >83.3 points; sufficient 66.7–83.3; problematic 50.1–66.6; inadequate ≤50) [6].

**TABLE 1:** Sample characteristics (Germany, 2022; N = 2,201^a^).

Characteristic (n missing)	n (%)
Age categories	
18–40	721 (32.7)
41–59	743 (33.7)
≥60	737 (33.5)
Sex	
Male	1,077 (48.9)
Female	1,124 (51.1)
Migration history (37)	
No	1,670 (77.2)
1st generation	159 (7.4)
2nd generation	335 (15.5)
Income (326)	
1st quartile (highest)	471 (25.1)
2nd quartile	466 (24.9)
3rd quartile	481 (25.7)
4th quartile (lowest)	457 (24.3)
Education (64)	
Highest	459 (21.5)
High	367 (17.2)
Intermediate	655 (30.6)
Low	656 (30.7)
Health insurance (8)	
Statutory	1,921 (87.6)
Private	272 (12.4)
Navigational health literacy (242)	
Inadequate	1,371 (70.0)
Problematic	117 (6.0)
Sufficient	162 (8.3)
Excellent	309 (15.8)
Unmet needs in the past 12 months (273^b^)	
Yes	588 (30.5)
No	1,340 (69.5)

^a^ weighted.

^b^ including respondents who did not need medical care in the past 12 months.

Unmet needs were assessed as follows: Participants were first asked whether they had experienced a need for examination or treatment in the past 12 months. If not, participants were excluded from the analyses. Overall, 273 participants were excluded from the unmet needs variable because they reported no need for medical care in the past 12 months or had missing information on unmet needs (see Table 1). Subsequently, three questions relating to forgone care addressed the most relevant, system-related reasons for unmet needs [29]: [1] “During the past 12 months did it ever happen that you did not get the medical treatment you needed *because you could not pay for it*?” [2], “During the past 12 months did it ever happen that you did not get the medical treatment you needed *because the treatment was not available where you live or nearby*?”, and [3] “During the past 12 months did it ever happen that you did not get the medical treatment you needed *because the waiting time/waiting list was too long*?” Response options were “yes”, “no”, “don’t know”/”not specified”. A composite variable was created indicating whether at least one of these types of unmet needs had been experienced in the past 12 months.

### Analyses

First, the percentage distribution of navigational health literacy categories and the prevalence of unmet needs were calculated. Bivariate associations between social characteristics, navigational health literacy, and unmet needs were primarily described using percentages. Pearson´s chi-square tests were used as supplementary overall tests of association between each social characteristic and the respective outcome variable. The association between navigational health literacy and unmet needs was additionally visualized in Figure 1. Finally, multiple Poisson regression models with log link and robust standard errors were conducted to estimate adjusted prevalence ratios (aPR) for unmet needs. Model 1 included social characteristics as predictors, while Model 2 additionally included navigational health literacy to examine whether adjustment for navigational health literacy attenuated the associations between social characteristics and unmet needs. All analyses were performed using the survey weights. Adjusted prevalence ratios, 95%-confidence intervals (95%-CI), and p-values were documented. Participants with missing values in any model variable were excluded. Statistical analyses were performed using the program package SPSS 29 [30].

**FIGURE 1:**
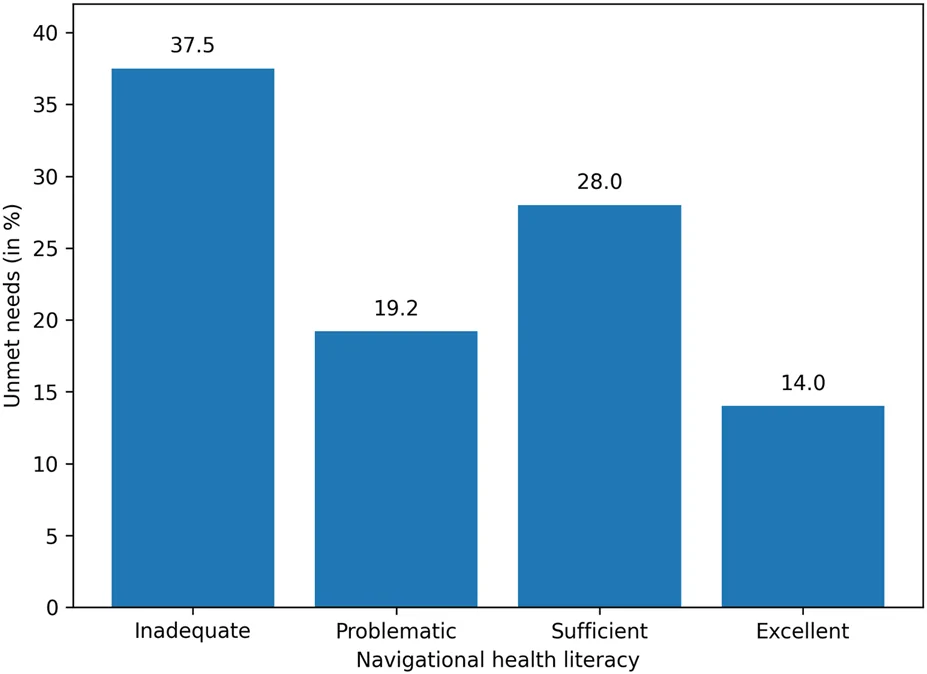
Prevalence of unmet needs across navigational health literacy categories (Germany, 2022; p < 0.001). The p-value refers to the overall Chi^2^-Test of association between navigational health literacy and unmet needs.

## Results

Table 1 presents the distribution of the social characteristics, the prevalence of self-reported unmet needs (30.5%), and the levels of navigational health literacy in the analyzed sample. Within this sample, approximately 70% of the participants exhibited inadequate, 6% problematic, 8.3% sufficient, and 15.8% excellent navigational health literacy.

In Table 2, social disparities in navigational health literacy are shown. The largest differences were observed by age, income, education, and health insurance status, whereas differences by sex were minimal. Participants aged 41–59 years had the highest proportion of inadequate navigational health literacy (76.0%), while those aged ≥60 years had the lowest proportion of inadequate navigational health literacy (64.4%) and the highest proportion of excellent navigational health literacy (21.1%). Differences by income showed a social gradient, with inadequate navigational health literacy being more common in the two lower income quartiles than in the two higher income quartiles. Similarly, participants with the highest educational level had a lower proportion of inadequate navigational health literacy (62.7%) than those with low education (72.7%). Differences were also observed by health insurance status, with privately insured participants showing a lower proportion of inadequate navigational health literacy (60.1%) and a higher proportion of excellent navigational health literacy (22.6%) than statutorily insured participants.

**TABLE 2:** Social disparities in navigational health literacy, in % (Germany, 2022; n = 1,693–1,960^a^).

Characteristic	Inadequate navigational health literacy	Problematic navigational health literacy	Sufficient navigational health literacy	Excellent navigational health literacy
Age categories	p < 0.001	​	​	​
18–40	69.4	8.3	8.6	13.6
41–59	76.0	4.0	7.3	12.6
≥60	64.4	5.7	8.8	21.1
Sex	p = 0.827	​	​	​
Male	70.4	6.0	7.6	16.0
Female	69.7	6.0	8.8	15.6
Migration history	p = 0.047	​	​	​
No	69.7	6.5	7.5	16.3
1st generation	69.9	3.4	8.9	17.8
2nd generation	71.2	5.0	12.0	11.7
Income	p = 0.020	​	​	​
1st quartile	63.8	6.9	8.0	21.3
2nd quartile	65.0	6.8	10.0	18.2
3rd quartile	72.8	6.9	8.0	12.3
4th quartile	72.6	5.3	8.1	14.1
Education	p = 0.010	​	​	​
Highest	62.7	7.7	13.1	16.5
High	69.1	6.9	7.2	16.9
Intermediate	71.6	5.1	7.9	15.4
Low	72.7	5.5	6.6	15.2
Health insurance	p = 0.003	​	​	​
Statutory	71.3	5.8	8.0	14.9
Private	60.1	7.0	10.3	22.6

^a^ weighted.

The p-values refer to overall Chi^2^-Tests of association between each social characteristic and navigational health literacy.

Table 3 displays bivariate associations between social characteristics and unmet needs. Differences were most pronounced by age, sex, income, and health insurance status. The prevalence of unmet needs was highest among participants aged 18–40 years (38.4%) and lowest among those aged ≥60 years (21.5%). Women reported unmet needs more often than men (35.9% vs. 24.8%). A clear income gradient was observed, with unmet needs increasing from 19.8% in the highest income quartile to 39.6% in the lowest income quartile. Differences were also observed by health insurance status, with unmet needs being more common among statutorily insured participants than among privately insured participants (32.6% vs. 16.0%). By contrast, differences by migration history and education were small.

**TABLE 3:** Prevalence of unmet needs by social characteristics, in % (Germany, 2022).

Characteristic	Unmet needs, in % (n = 1,650 −1,928^a^)
Age categories	p < 0.001
18–40	38.4
41–59	32.7
≥60	21.5
Sex	p < 0.001
Male	24.8
Female	35.9
Migration history	p = 0.851
No	30.6
1st generation	29.0
2nd generation	31.6
Income	p < 0.001
1st quartile	19.8
2nd quartile	30.4
3rd quartile	32.1
4th quartile	39.6
Education	p = 0.630
Highest	28.0
High	30.5
Intermediate	30.5
Low	32.0
Health insurance	p < 0.001
Statutory	32.6
Private	16.0

^a^ weighted.

The p-values refer to overall Chi^2^-Tests of associations between each characteristic and unmet needs.

To further illustrate the association between navigational health literacy and unmet needs, Figure 1 displays the prevalence of unmet needs across navigational health literacy categories. The prevalence of unmet needs was highest among participants with inadequate navigational health literacy (37.5%) and lowest among those with excellent navigational health literacy (14.0%), with an overall statistically significant association between navigational health literacy and unmet needs (p < 0.001).

Table 4 presents multiple Poisson regression models examining associations between social characteristics, navigational health literacy, and unmet needs using adjusted prevalence ratios. In Model 1, older age and private health insurance were associated with a lower adjusted prevalence ratio of unmet needs, whereas female sex and lower income were associated with higher adjusted prevalence ratios for unmet needs. Migration history and education showed no clear correlation with unmet needs. After adding navigational health literacy to Model 2, the prevalence ratios for social characteristics remained largely unchanged. Compared to respondents with an inadequate navigational health literacy, those with a problematic navigational health literacy (aPR: 0.55; 95%-CI 0.37–0.80) and excellent navigational health literacy (aPR: 0.38; 95%-CI: 0.27–0.53) had a lower prevalence of unmet needs.

**TABLE 4:** Associations of social characteristics and navigational health literacy with unmet needs: adjusted prevalence ratios and 95%-confidence intervals from multiple Poisson regression models (Germany, 2022; n = 1,552^a^).

​	​	Unmet needs	
Characteristic	Category	Model 1	Model 2
Age categories	41–59	0.79 (0.67–0.94)**	0.78 (0.66–0.93)**
​	≥60	0.57 (0.46–0.71)***	0.61 (0.49–0.75)***
Sex	Female	1.30 (1.13–1.51)***	1.32 (1.14–1.52)***
Migration history	1st generation	1.12 (0.88–1.44)	1.10 (0.86–1.41)
​	2nd generation	1.01 (0.83–1.23)	0.99 (0.82–1.20)
Income	2nd quartile	1.42 (1.12–1.80)**	1.41 (1.11–1.78)**
​	3rd quartile	1.52 (1.19–1.93)**	1.45 (1.15–1.84)**
​	4th quartile	1.67 (1.33–2.12)***	1.62 (1.29–2.04)***
Education	High	0.83 (0.64–1.07)	0.83 (0.65–1.07)
​	Intermediate	0.99 (0.81–1.21)	0.96 (0.78–1.17)
​	Low	1.08 (0.86–1.36)	1.03 (0.82–1.29)
Health insurance	Private	0.60 (0.43–0.82)**	0.62 (0.45–0.84)**
Navigational health literacy	Problematic	-	0.55 (0.37–0.80)**
​	Sufficient	-	0.77 (0.59–1.00)
​	Excellent	-	0.38 (0.27–0.53)***

^a^ weighted.

Reference categories: Age: 18–40 years, Sex: Male, Migration history: No, Income: 1st quartile (highest), Education: Highest, Health insurance: Statutory, Navigational health literacy: Inadequate.

* = p < 0.05, ** = p < 0.01, *** = p < 0.001; all variables in the models are mutually adjusted. Bold values indicate statistically significant associations (p < 0.05).

## Discussion

Our findings revealed that a substantial proportion of the German adult population reported inadequate navigational health literacy (70%). Bivariate analyses showed that lower navigational health literacy was particularly common among participants aged 41–59 years, as well as among participants with lower income, lower education, and statutory health insurance, and that inadequate navigational health literacy was strongly linked to higher unmet needs. In multiple Poisson regression models, navigational health literacy was associated with a lower adjusted prevalence of unmet needs. However, we did not observe a linear association between the individual categories of navigational health literacy and the frequency of unmet needs. Specifically, the sufficient navigational health literacy category, unlike the problematic and excellent categories, was not significantly associated with a lower prevalence of unmet needs. This may be due to the uneven distribution of participants across categories, with only 8.3% classified as having sufficient navigational health literacy. Finally, navigational health literacy did not attenuate the observed social disparities in unmet needs.

Our study indicated rates of inadequate navigational health literacy that are consistent with findings from comparable German studies [6, 10]. Notably, navigational health literacy appeared to be lower than other components of health literacy, such as digital and communicative health literacy [6], suggesting that individuals face particularly pronounced difficulties in this domain. The fact that 70% of participants fell into the inadequate category underscores the urgent need for intervention. Our results highlighted social disparities in navigational health literacy, as certain socio-demographic groups demonstrated significantly lower levels. Specifically, individuals with statutory health insurance, low income, low education, and aged 41–59 years showed significantly lower navigational health literacy. These findings align with previous research suggesting social inequalities in navigational health literacy [6, 10, 12]. International evidence indicates that both navigational health literacy and unmet needs are socially patterned beyond the German healthcare system. In the HLS19-NAV validation study conducted in eight European countries, navigational health literacy was associated with socioeconomic factors across countries. Financial deprivation and self-perceived social status were significant predictors in seven of the eight countries. Moreover, navigational health literacy was a significant predictor of self-reported general health status in seven of the eight countries [11]. International studies on unmet needs point in a similar direction. Analyses across EU and OECD countries found that more than a quarter of adults with a need for care reported barriers due to costs, waiting times, or distance, with substantial variation between countries and a clear social gradient disadvantaging lower-income groups [16, 31]. Germany was among the countries with comparatively high income-related inequalities in unmet needs, which is consistent with the social patterning observed in our study [16].

Adding to this evidence, our study found that low navigational health literacy was significantly associated with a higher prevalence of unmet needs. To our knowledge, this is the first study to investigate this correlation, further highlighting the critical role of health literacy in determining healthcare access. Importantly, the adjusted prevalence ratios for social characteristics and unmet needs remained largely unchanged after introducing navigational health literacy into the model. These results suggest that navigational health literacy was independently associated with unmet needs but did not substantially reduce the observed social disparities in unmet needs. These findings indicate that strengthening navigational health literacy alone is unlikely to eliminate disparities in access to care unless structural barriers are also addressed.

Our findings underline the need for targeted interventions to strengthen navigational health literacy in the German population, with particular focus on deprived social groups. Previous intervention efforts [1, 4, 32] have not yet achieved sufficient improvements, as demonstrated by our study, suggesting that current strategies may not adequately address the underlying barriers. Many existing programs primarily focus on providing general health information. To improve individuals’ ability to navigate in complex healthcare systems, it could be beneficial to focus more on developing specific navigational skills, such as accessing healthcare information and resources, identifying appropriate health services, managing administrative and organizational tasks, understanding patients’ rights, and advocating for them. Consequently, more targeted, population-specific interventions could help address existing inequalities more effectively.

Since research has shown that delayed or forgone care is associated with adverse health outcomes [24, 33–36], it is crucial to establish effective interventions to reduce its prevalence. Given that both navigational health literacy and unmet needs in our study are linked to similar socioeconomically disadvantaged populations, these groups should be the primary focus of targeted efforts to mitigate potential health risks and promote equity in healthcare access. Strengthening individuals’ ability to navigate the healthcare system can play a key role in reducing unmet needs and ultimately preventing health deterioration. However, our findings also suggest that difficulties in navigating healthcare systems are not confined to those with limited education or financial resources. Even individuals with high income, education, or private health insurance showed low navigational health literacy, indicating that challenges in this domain are at least partly rooted in structural deficiencies. As such, improving navigational health literacy should not be limited to promoting individual competencies but must be accompanied by systemic reforms.

Practical strategies to improve navigational health literacy include the provision of accessible, reliable, and easy-to-understand information essential for orientation within the healthcare system. Healthcare facilities should implement clear wayfinding systems, user-centered signage, and provide contact persons or patient navigators to support individuals throughout their care journey [10, 37]. In this regard navigational health literacy is closely aligned with the concept of organizational health literacy, which Brach defines as *“the degree to which an organization implements policies, practices, and systems that make it easier for people to navigate, understand, and use information and services to take care of their health”* [38]. On a broader level, the healthcare system should strive to become a health-literate environment: transparent, intuitive, and easy to navigate for all patients [39, 40]. Achieving this goal requires institutional commitment and the training of healthcare professionals to act as facilitators of navigation. Additionally, embedding navigational health literacy training into school curricula, adult education programs, and digital health platforms could be effective long-term strategies. System-level changes should be accompanied by structural health literacy audits within institutions to identify and reduce barriers to navigation.

While improving navigational health literacy is essential, it must go hand in hand with efforts to address systemic healthcare barriers to sustainably reduce disparities. Future research should investigate the causal pathways between navigational health literacy and unmet needs through longitudinal designs and evaluate the effectiveness of both individual- and system-level interventions tailored to the needs of disadvantaged populations. Addressing navigational health literacy as part of a comprehensive approach to health equity may be a key to achieving accessible healthcare for all.

### Limitations

There are some limitations that must be considered when interpreting our results. The analyses were based on an online survey, and although the sample was randomly drawn from a panel recruited offline, participation was limited to individuals with internet access. Moreover, selection bias cannot be ruled out, as only approximately 39% of invited individuals participated. To mitigate this potential bias, the data were weighted for age, sex, federal state, and education according to official statistics using an iterative proportional fitting approach [26, 27]. However, weighting cannot account for unobserved differences between participants and non-participants, such as health status, healthcare experiences, digital access, or interest in health-related topics. Furthermore, the analyses were restricted to individuals proficient in reading German. This is particularly important when interpreting results related to language barriers and migration history. Consequently, associations with migration history may have been underestimated. Similarly, the impact of language barriers on navigational health literacy and unmet needs may have been underestimated. Another limitation concerns missing values. While the full sample included 2,201 participants, both multiple Poisson regression models were based on the same analytical sample of 1,552 respondents, excluding participants with missing values in any model variables. If excluded participants differed systematically from those included, the estimated associations may be biased. Because missingness was most pronounced for income and navigational health literacy, the exclusion of participants with missing values may have led to an underrepresentation of socioeconomically disadvantaged respondents or respondents with lower navigational health literacy. This may have resulted in conservative estimates of social disparities. Regarding navigational health literacy, it is important to consider that it was self-assessed using the HLS19-NAV instrument. The limitations of self-assessment instruments, such as the potential for over- or underestimation, are documented in the literature [41]. Similarly, unmet needs were also self-reported by the participants. Thus, recall bias due to the 12-month recall period, which may have affected the accuracy of participants' self-reported information, and common-method bias, resulting from the assessment of key variables using the same self-report method, cannot be ruled out. The categorization of navigational health literacy was based on previous studies [6]. However, the distribution across the different categories was uneven, with 70% of the participants falling into the inadequate category. This imbalance may have contributed to certain associations not reaching statistical significance, for instance, the association between sufficient navigational health literacy and lower levels of unmet needs. It may also have limited the ability to detect gradients within the lower range of navigational health literacy. An additional factor to note is that the cross-sectional design of the study precludes any inferences regarding direction of causality. Therefore, it remains unclear whether limited navigational health literacy contributes to unmet needs, whether unmet needs affect perceived navigational health literacy, or whether both are influenced by other individual or structural factors.

### Conclusions

A substantial proportion of the participants demonstrated low navigational health literacy. While navigational health literacy did not attenuate social disparities in unmet needs in this study, it was significantly associated with unmet needs itself. Moreover, both unmet needs and navigational health literacy showed significant associations with social characteristics, highlighting the inequality in healthcare access. Given that low navigational health literacy is correlated with a higher prevalence of unmet needs, which in turn may contribute to a worsening in health status [24, 33–36], it is essential to implement further targeted interventions aimed at improving navigational health literacy. These efforts should specifically focus on socioeconomically disadvantaged groups, as they are disproportionally affected. Improving navigational health literacy within these populations is essential not only for ensuring equitable healthcare access but also for reducing overall health disparities.
